# Identification of ebselen and its analogues as potent covalent inhibitors of papain-like protease from SARS-CoV-2

**DOI:** 10.1038/s41598-021-83229-6

**Published:** 2021-02-11

**Authors:** Ewelina Weglarz-Tomczak, Jakub M. Tomczak, Michał Talma, Małgorzata Burda-Grabowska, Mirosław Giurg, Stanley Brul

**Affiliations:** 1 Molecular Biology and Microbial Food Safety Group, Swammerdam Institute for Life Sciences, Faculty of Science, University of Amsterdam, Amsterdam, The Netherlands; 2 Computational Intelligence Group, Department of Computer Science, Faculty of Science, Vrije Universiteit Amsterdam, Amsterdam, The Netherlands; 3 Department of Bioorganic Chemistry, Faculty of Chemistry, Wroclaw University of Science and Technology, Wrocław, Poland; 4 Department of Organic and Medicinal Chemistry, Faculty of Chemistry, Wroclaw University of Science and Technology, Wrocław, Poland

**Keywords:** Enzymes, Proteases, Molecular modelling, Medicinal chemistry, Infectious diseases, Screening, Small molecules

## Abstract

An efficient treatment against a COVID-19 disease, caused by the novel coronavirus SARS-CoV-2 (CoV2), remains a challenge. The papain-like protease (PL^pro^) from the human coronavirus is a protease that plays a critical role in virus replication. Moreover, CoV2 uses this enzyme to modulate the host’s immune system to its own benefit. Therefore, it represents a highly promising target for the development of antiviral drugs. We used Approximate Bayesian Computation tools, molecular modelling and enzyme activity studies to identify highly active inhibitors of the PL^pro^. We discovered organoselenium compounds, ebselen and its structural analogues, as a novel approach for inhibiting the activity of PL^pro^CoV2. Furthermore, we identified, for the first time, inhibitors of PL^pro^CoV2 showing potency in the nanomolar range. Moreover, we found a difference between PL^pro^ from SARS and CoV2 that can be correlated with the diverse dynamics of their replication, and, putatively to disease progression.

## Introduction

A serious respiratory disease caused by severe acute respiratory syndrome (SARS) coronavirus has been reported three times in the last 20 years. It was first identified during the 2002–2003 SARS outbreak (SARS-CoV-1) that resulted in 8422 reported cases with a case fatality rate (CFR) of 11%^1^. One decade after SARS-CoV-1, a new coronavirus, Middle East respiratory syndrome coronavirus (MERS-CoV), had been established with CFR of as much as 43%^2^. Due to high fatality and the implementation of infection control measures, MERS-CoV outbreak was successfully ended. In December 2019, a novel coronavirus, SARS coronavirus 2 (SARS-CoV-2, CoV2), was discovered, and further sequenced and isolated by January 2020^3,4^. Due to a relatively long incubation period, and mixed symptoms with different levels of severity, CoV2 has spread world-wide, infecting over 40 million people, and caused over 1.1 million deaths^5^. These facts about SARS-CoV-1, MERS, and CoV2 clearly indicate how fatal the interspecies transmission potential of coronaviruses is.

The development of anti-coronaviral drugs remains challenging although a number of coronaviral proteins have been identified as potential drug targets against SARS^6,7^ and recently CoV2^8,9^. Both viruses strongly rely for their replication in particular on the viral Papain-like protease (PL^pro^)^10,11^. This enzyme, next to the Main Protease (M^pro^, also known as chymotrypsin-like protease 3CLpro)^12^, plays an essential role in polypeptide processing. PL^pro^ from SARS had been further characterised as a protease that recognizes the P4–P1 consensus cleavage sequence LXGG, found at the boundaries of nsp1/2, nsp2/3 and nsp3/4 where membrane association is required for cleavage of the nsp3/4^11,13,14^. PL^pro^, as a result of being crucial during replication via processing of the viral polyprotein^11^, was proposed to be a key enzyme in the sustained pathogenesis of SARS-CoV^15–18^. This includes deubiquitination (the removal of ubiquitin), and deISGylation (the removal of ISG15) from host-cell proteins and results in the antagonism of the host antiviral innate immune response^14–16^.

Very recent studies led by Dikic confirmed the PL^pro^ from novel coronavirus CoV2 (PL^pro^CoV2) to be an essential viral enzyme and potentially its weak spot^19^. They proposed PL^pro^ to be Achilles’ heel of SARS-CoV-2. PL^pro^ from SARS-CoV-1 (PL^pro^SARS) and PL^pro^CoV2 are strongly related, with 82.9% sequence identity, and relatively distant from PL^pro^ from MERS (32.9% identity). Notwithstanding their similarity, PL^pro^CoV2 and PL^pro^SARS differ in their specificity toward cleaving ISG15 and ubiquitin chains. PL^pro^CoV2 predominantly cleaves the ubiquitin-like protein ISG15 from host proteins and, therefore, possesses a higher ability to block the type 1 IFN pathway that is correlated with the initiation of antiviral innate immunity. PL^pro^SARS preferentially targets ubiquitin chains and suppresses more preferentially the NF-κB pathway that is responsible for initiation of cell death and strong pro-inflammatory cytokine response^19^. Independent research led by Drag, in which synthetic fluorogenic substrate library was used, also confirmed the P4–P1 consensus cleavage sequence LXGG to be recognized by PL^pro^^20^. As a result, PL^pro^ is an important potential target for antiviral drugs that may inhibit viral replication and reduce dysregulation of immune response.

Ebselen is a low-molecular-weight organoselenium drug that shows a pleiotropic mode of action. Due to its very low toxicity there are no barriers to using it in humans^21^. It is a well-known agent with therapeutic activity in neurological disorders^22^ and cancers^23^. Ebselen also showed an antiviral effect on neurotropic viruses^24^, the hepatitis C virus^25^, the human immunodeficiency virus^26–28^, and other virucidal and antimicrobial activity^29,30^. Recently, ebselen has been shown to attenuate inflammation and promote microbiome recovery in mice after antibiotic treatment for CDIAs^31^. Several lines of evidence demonstrated the biological effects of ebselen is mainly due to its antioxidant properties and capability of forming selenenyl-sulfide bonds with the cysteine residues in proteins^22,32–34^. In recently published work, ebselen has been found, through a combination of structure-based virtual and high-throughput screening, as an effective inhibitor of M^pro^ from CoV2 showing half-maximal inhibitory concentration value (*IC*_50_) equal 0.67 µM and also exhibiting promising antiviral activity in cell-based assay (*EC*_50_ = 4.67 µM)^35^.

Here, we demonstrate that ebselen inhibits activity of the PL^pro^ from CoV2 in a low micromolar range. We have identified the mechanism of inhibition as fast and irreversible as well as propose the binding mode of ebselen by molecular docking.

Furthermore, we report here inhibitory activity of seven ebselen derivatives/analogues functionalization at position 2 of benzisoselenazol-3(*2H*)-one ring and four related bis(2-carbamoyl)phenyl diselenides towards PL^pro^SARS and PL^pro^CoV2. Similarly, to ebselen, all analogues show irreversible modes of action. The SAR studies led to the identification of four strong inhibitors that bind favorably to the PL^pro^CoV2 with the half maximal inhibitory concentration in the nanomolar range. They are first as potent inhibitors of PL^pro^ from CoV2 reported so far, and, therefore, may contribute to the development of drugs against COVID-19. Moreover, we have found a difference in the mechanism of catalysis, the dynamics of inhibition and the active sites between PL^pro^ from SARS and CoV2.

Our results, of great significance for the development of effective drugs against COVID-19, open a new approach to fight the still spreading pandemic disease.

## Results

### New PL^pro^ inhibitors are proposed via analysis of the active site

**Figure 1:**
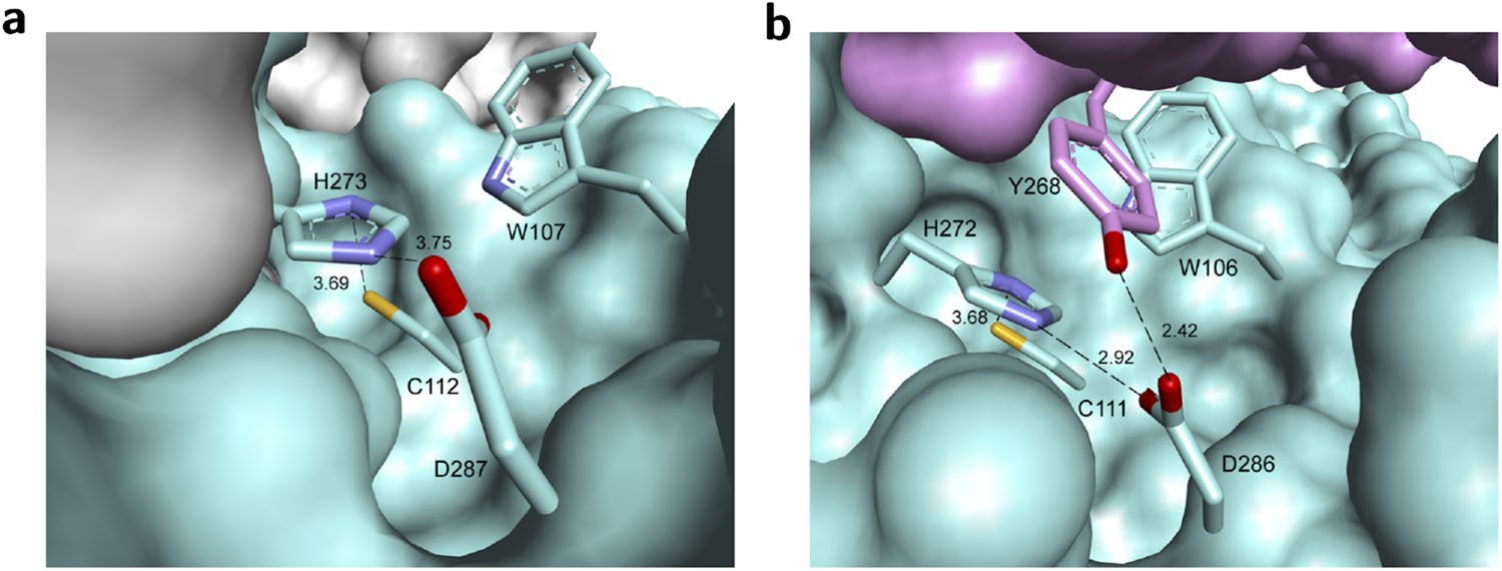
The active site of PL^pro^s contains a classic catalytic triad, composed of Cys–His–Asp, where Cys acts as nucleophile. (**a**) Unliganded catalytic center of the active site of PL^pro^SARS (PDB: 2FE8^10^). (**b**) Unliganded catalytic center of the active site of PL^pro^CoV2 (PDB: 6W9C^37^).

We analyzed the active site and the mechanism of PL^pro^ from SARS based on the crystal structure published by Báez-Santos et al.^10,36^ that showed PL^pro^SARS has a catalytic triad composed of Cys112–His273–Asp287 (Fig. 1a). This catalytic triad transforms the –SH group from cystine into a strong nucleophilic ion that attacks the carboxylic group of peptide bonds and leads to hydrolysis (Supplementary Fig. S1 (left)). The side chain sulfur atom of Cys112 is positioned 3.7 Å from the nitrogen in position 1 of the imidazole ring in the catalytic histidine (His273). One of the oxygen atoms of the side chain of the catalytic aspartic acid (Asp287) is located 3.7 Å from the nitrogen in position 3 of the same histidine (Fig. 1a). The side chain of Trp107 that is located within the oxyanion cavity participates in the stabilization of the negatively charged tetrahedral transition state of the reaction intermediates produced throughout catalysis^10,17,36^.

Further, we analyzed the crystal structure of PL^pro^ from CoV2 published recently in the PDP database by Osipiuk et al.^37^. Apparently, PL^pro^ from CoV2 displays a different conformation compared to PL^pro^SARS. In PL^pro^SARS the catalytic triad is exposed externally at the outer surface of the protein. Whereas, in PL^pro^CoV2 the Cys111–His272–Asp286 motif in each of three subunits is directed towards the center of the protein (Supplementary Fig. S2). The distance between the sulfur on Cys111 and nitrogen 1 of the imidazole ring in His272 is almost the same as it is in PL^pro^SARS. While one of the oxygen atoms of the side chain of the catalytic aspartic acid (Asp286) is located 2.9 Å from the nitrogen in position 3 of the same histidine (Fig. 1b), it is 0.8 Å closer than in PL^pro^SARS. Moreover, in the PL^pro^CoV2 Tyr268 appears closer to the active site forming a hydrogen bond with the second oxygen from the side chain of Asp287.

The analysis of the active site and mechanism of catalysis of coronaviruses PL^pro^ (Fig. 1; Supplementary Fig. S1) led us to conclude that small molecules with planar phenyl moieties, which are also able to modify a cysteine residue in the active site, could be effective. The seleno-organic drug ebselen meets such active site requirements. Its size, conformation, and the ability to modify the -SH group makes this compound a perfect candidate for inhibition of PL^pro^.

**Figure 2:**
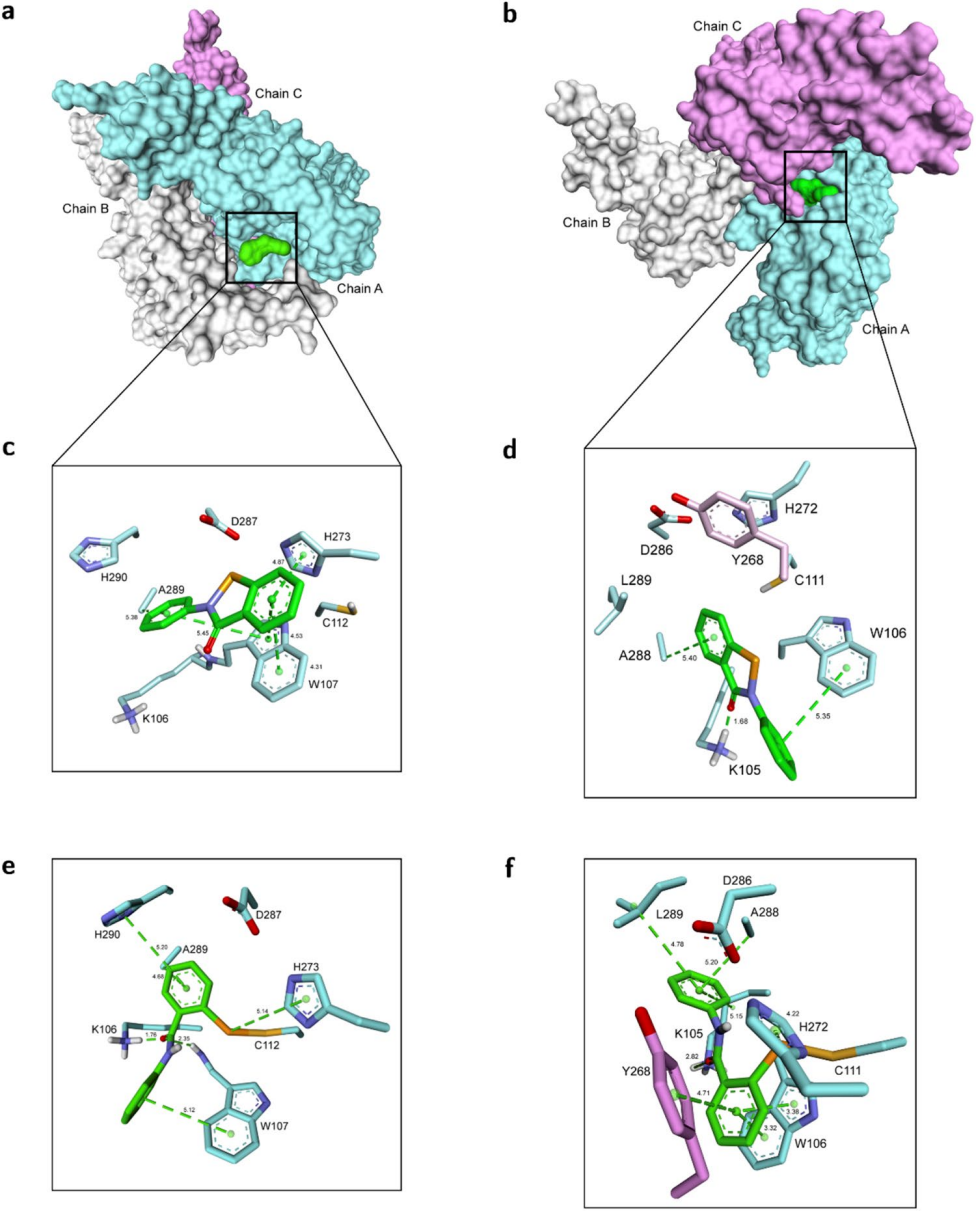
Ebselen is buried deeper in PL^pro^CoV2 than in PL^pro^SARS. (**a**,**b**), Molecular surface representation of the multi-domain architecture of PL^pro^SARS (PDB: 2FE8^10^) (**a**) and PL^pro^CoV2 (PDB: 6W9C^37^) (**b**) in complex with ebselen. (**c**,**d**), Stick representation of the interactions between ebselen and residues in the active site of PL^pro^SARS (**c**) and PL^pro^CoV2 (**d**) before covalent modification of cysteine residue**.** (**e**,**f**) Stick representation of the interactions between ebselen and residues in the active site of PL^pro^SARS (**e**) and PL^pro^CoV2 (**f**) after covalent modification of cysteine residue.

Our hypothesis was further illustrated by the use of molecular modeling to study possible binding modes of ebselen with PL^pro^SARS (Fig. 2a,c,e) and PL^pro^CoV2 (Fig. 2b,d,f). The molecular docking seemed to confirm our primary assumption that ebselen can interact with the PL^pro^CoV2 active site and, thus, convinced us of our hypothesis about ebselen as a possible active site binding inhibitor. In order to recognize what happens before hypothesized covalent modification of the catalytic cysteine, we performed the molecular modeling study on the binding mode of the unreacted ebselen with both proteases. The models with the most favorable thermodynamic stability show that ebselen occupies an intersection between the putative catalytic triad and tryptophan, namely, Cys112–His273–Asp287 and Trp107 for PL^pro^SARS, and Cys111–His272–Asp286 and Trp106 for PL^pro^CoV2, as we hypothesized (Fig. 2c,d). In PL^pro^SARS, the *N*-phenyl ring of ebselen is surrounded by the aromatic ring of Trp107 (edge-to-face interaction) and Ala289. Whereas, the *Se*-ring forms π–π interaction with the indole ring of Trp107 and edge-to-face interaction with His273 (Fig. 2c). In the case of PL^pro^CoV2, the *N*-phenyl group is directed toward intersection between Trp106 and Lys105, while the *Se*-ring interacts with Ala 288 (π-alkyl). Whereas, the C=O group binds with the side chain –NH_2_ of Asp105. The H⋯O distance is 1.68 Å, revealing a tight hydrogen bonding interaction (Fig. 2d).

Further, we modeled the ebselen–PL^pro^SARS and ebselen–PL^pro^CoV2 adducts resulting from the reaction of ebselen with Cys111 and Cys112, respectively (Fig. 2d,e). The fragment derived from the ebselen molecule fit the region near the active site, and its positioning completely blocked the entrance to the active site. The most preferable conformation for the ebselen–PL^pro^SARS covalent complex shows a slightly different arrangement then noncovalent one. The *N*-phenyl ring of ebselen is still surrounded by the aromatic ring of Trp107 but it is not anymore directed toward Ala289. The amine group from Lys106 additionally interacts with oxygen from ebselen. We identified another edge-to-face interaction, i.e., between the *Se*-ring and an imidazole side chain of His290 (Fig. 2e).

A deeper penetration of PL^pro^CoV2 than of PL^pro^SARS by ebselen can be observed for covalent complexes (Fig. 2b,d). This effect results from a different conformation of the enzyme and from the fact that the active site in PL^pro^SARS is exposed on the outer surface of the protein and in PL^pro^CoV2 it is directed towards the center of the protein. Moreover, ebselen is additionally wrapped by other amino acids (Tyr268, Ala288, Leu289) (Fig. 2f). Here, we identified a different binding mode where the *Se*-phenyl is directed to the oxyanion hole and Trp106, not the *N*-phenyl as was observed for PL^pro^SARS (Fig. 2e,f). The *Se*-phenyl (from ebselen) and an indole (from Trp106) formed π–π stacking interactions, while face-to-edge stacking interactions were observed with an aromatic ring from Tyr268. The *N*-phenyl ring of ebselen adopts a bent-shaped conformation that fits well to the space between Leu298 and Ala288 forming π-alkyl interaction with them. The tight hydrogen interaction between Lys105 and C=O from unreacted ebselen (Fig. 2d) seems to be important factor for stabilization of the complex and could contribute to the rate of the covalent reaction between Se and SH group from Cys111.

### Kinetic analysis of the Ub-AMC hydrolysis by PL^pro^ from SARS and CoV2 show differences in catalytic efficiency

**Figure 3:**
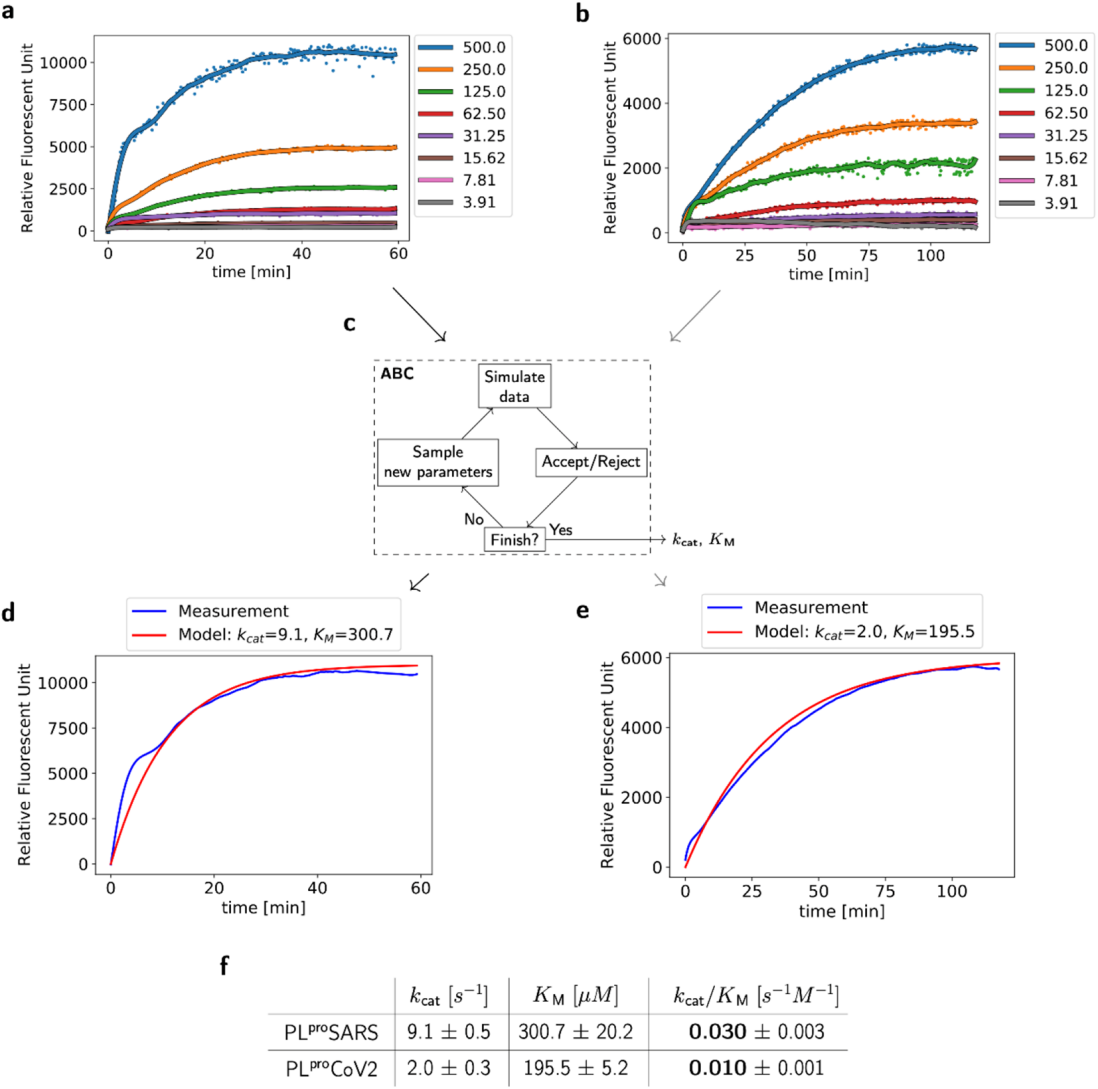
PL^pro^SARS displays a higher catalytic efficiency than PL^pro^CoV2 toward Ub-AMC. (**a**,**b**) Progress curves for the hydrolysis of Ub-AMC by PL^pro^SARS (**a**) and PL^pro^CoV2 (**b**). The concentrations of Ub-AMC are shown in the legend in nM, PL^pro^SARS and PL^pro^CoV2 were10 nM. (**c**) Four steps of the Approximate Bayesian Computation (ABC) for calculating values of kinetic constants *k*_cat_ and *K*_M_. (**d**,**e**) A comparison of measurements (in blue) and solutions of the Michaelis–Menten model for given parameters of enzymatic constants found by the ABC method (in red) for PL^pro^SARS (**d**) and PL^pro^CoV2 (**e**) at 500 nM substrate concentration (*k*_cat_ and *K*_M_ are expressed in s^−1^ and μM, respectively). (**f**) The kinetic data (*k*_cat_, *K*_M_, *k*_cat_/*K*_M_) for the Ub-AMC substrate of PL^pro^SARS and PL^pro^CoV2.

As a substrate for our study we chose Ubiquitin conjugated with a fluorophore (Ub-AMC). Progress curves (Fig. 3a,b) for reactions at different levels of substrate concentration showed an interesting difference between the two enzymes. PL^pro^SARS catalyzed the reaction and achieved saturation faster than PL^pro^CoV2. We estimated kinetic parameters of hydrolysis of Ub-AMC (Fig. 3f) using the recently published novel Approximate Bayesian Computation (ABC) computational tool for calculating kinetic constants in the Michaelis–Menten equation^38^ (Fig. 3c–e), see the Supplementary Data for more details. This extremely useful framework gives us the opportunity to find the turnover number (*k*_cat_), the Michaelis Menten constant (*K*_M_) and, as a consequence, the catalytic efficiency of the enzyme (*k*_cat_/*K*_M_) without using high concentrations of Ub-AMC (Fig. 3).

The catalytic efficiency (*k*_cat_/*K*_M_) is used as a specificity constant to compare the relative rates of reactions. Here we show that this ratio is three times higher for PL^pro^SARS compared to PL^pro^CoV2 that indicates its higher capability to hydrolyze Ub-AMC. PL^pro^ is required for the processing of viral polypeptides and to modulate the host’s immune response, the higher efficiency may contribute to the fact that once infected, SARS was overall more aggressive and the disease developed faster.

### Ebselen inhibits PL^pro^CoV2 in the micromolar range

**Figure 4:**
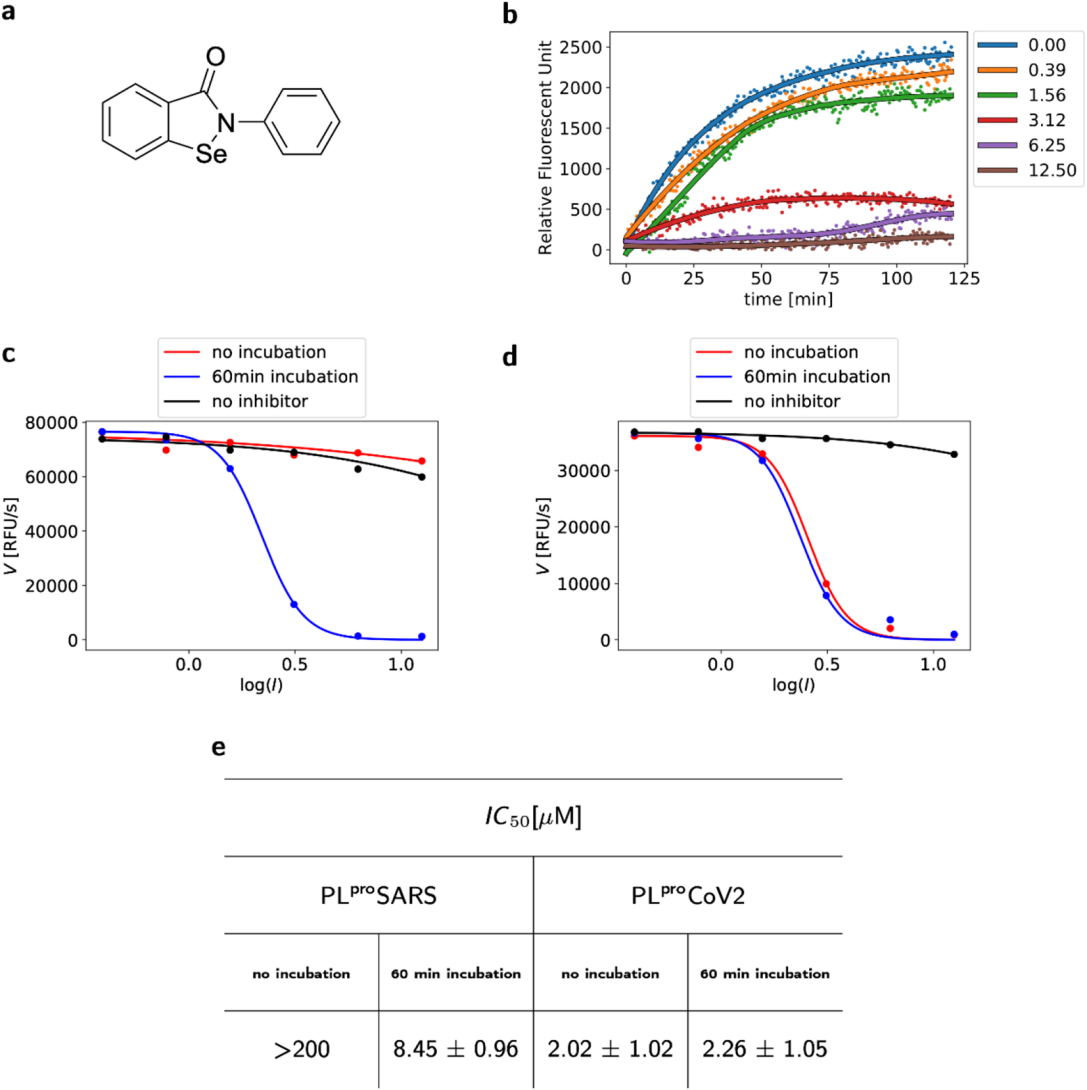
Ebselen inhibits PL^pro^CoV2 in the micromolar range. (**a**) Structure of ebselen. (**b**) Progress curves for the hydrolysis of Ub-AMC by PL^pro^CoV2 without and in the presence of increasing concentrations of ebselen. The concentrations of Ub-AMC and PL^pro^CoV2 were 500 nM and 10 nM, respectively, the concentration of ebselen is shown in the legend in μM. (**c**,**d**) PL^pro^CoV2 (**c**) and PL^pro^SARS (**d**) assay results for ebselen without incubation and after a 1-h incubation at 37 °C. Data points are plotted as the mean of *n* = 3 independent experiments, with each experiment having *n* = 1 independent samples. (**e**) Inhibitory activities of ebselen toward PL^pro^ from SARS and CoV2.

We applied ebselen as a possible inhibitor and, indeed, it suppresses PL^pro^ activity from CoV2 with inhibition constants approximately equal to 2 μM (Fig. 4). We determined the mechanism of inhibition of ebselen as irreversible, with steady-state binding being achieved immediately. Ebselen appeared to be an irreversible inhibitor of the studied PL^pro^SARS as well, although, in this case inhibition was slightly weaker and the kinetics of binding was slower, needing 60 min to achieve the most favourable *IC*_50_ value (Fig. 4e). We believe that slow binding mode observed for PLproSARS is caused by rearrangement of the enzyme and lower amount of the additional interactions between inhibitor and active site before and after covalent modification.

Irreversibility seems to confirm our first assumption that the inhibition of both enzymes can be associated with covalent bonds between –Se from ebselen and –S from cysteine. We then confirmed irreversibility via dialysis and via attempting to reactivate the enzymes (please see material and methods section). In case of reversible inhibitors, the recovery of activity could be accomplished by removing the ligand by dialysis. Here, the reactivation was not observed for enzymes treated by ebselen.

Slightly lower *IC*_50_ found for PL^pro^CoV2 can be explained by the smaller distance between the oxyanion hole and the ligand, as well as the greater number of interactions with the amino acids surrounding the active site (Fig. 2e,f)^37^.

### Structural analogues of ebselen as potent covalent inhibitors of PL^pro^CoV2

**Figure 5:**
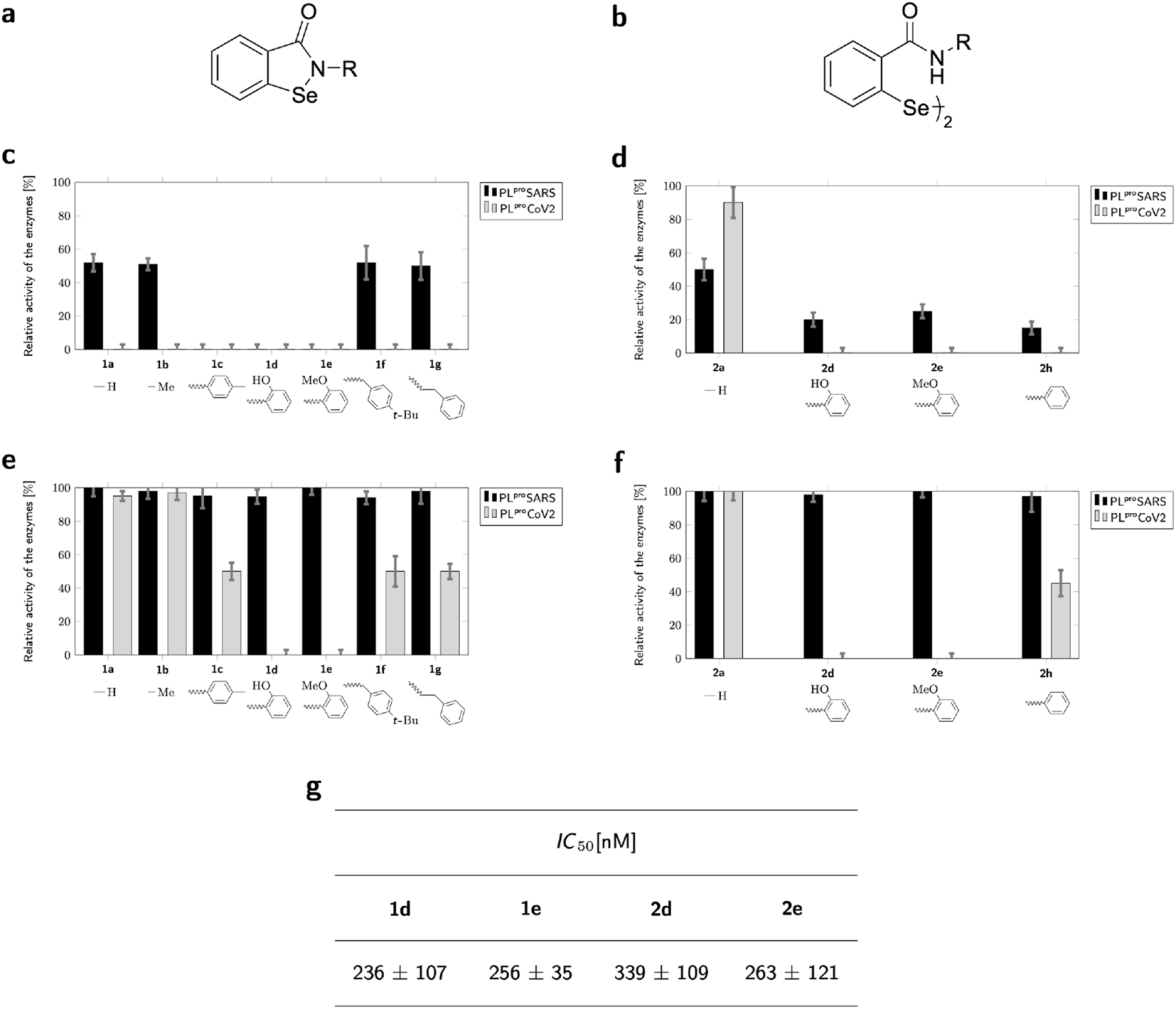
*N*-substituent optimization of ebselen leads to nanomolar potent inhibitors. (**a**,**b**) General structures of benzisoselenazol-3(2*H*)-ones **1a**–**1g** (**a**) and bis(2-carbamoyl)phenyl diselenides **2a**, **2d**, **2e**, **2h** (**b**) used in our study. (**c**,**d**) Relative activity of the PL^pro^SARS (black) and PL^pro^CoV2 (grey) in the presence of 20 μM of inhibitors being benzisoselenazolones **1a**–**1g** (**c**) and diselenides **2a**, **2b**, **2d** and **2e** (**d**). (**e**,**f**) Relative activity of the PL^pro^SARS and PL^pro^CoV2 in the presence of 2 μM of inhibitors being benzisoselenazolones **1a**–**1g** (**e**) and diselenides **2a**, **2b**, **2d** and **2e** (**f**). **g**, Inhibitory activity for selected ebselen derivatives substituted on the phenyl ring **1d** and **1e** and related diselenides **2d** and **2e** (the acyclic forms of **1d** and **1e**), respectively, toward PL^pro^CoV2.

Having established ebselen as an irreversible inhibitor of PL^pro^SARS and PL^pro^CoV2, we then investigated whether a modification of the lead compound can increase inhibitory potency. Eleven organoselenium compounds, ebselen derivatives/analogues, seven benzisoselenazol-3(2*H*)-ones (**1a**–**g**) (Fig. 5a,c,e) and four 2,2′-dicarbamoyldiaryl diselenides (**2a**, **2d**–**e**, **2h**) (Fig. 5b,d,f) were employed for inhibitory studies toward PL^pro^ from SARS and CoV2. The first group included 2-phenylbenzisoselenazol-3(2*H*)-ones with the phenyl ring replaced with H (**1a**) or Me(**1b**) or monosubstituted with a functional group, such as Me (**1c**), OH (**1d**), OMe (**1e**), and analogues of ebselen based on the benzisoselenazol-3(2*H*)-one core modified in position 2 (on the nitrogen atom) by adding –CH_2_– (**1f**) and –CH_2_CH_2_– (**1g**) linker (compound **1f** is additionally alkylated by *t*-Bu at 4 position of the phenyl ring). The second group constitutes the acyclic ebselen form **2h** or its derivatives **2d** and **2e** containing two atoms of selenium per molecule, known as their ‘dimeric’ forms. Organoselenium compounds show irreversibility of their inhibitory mode of action as well. All phenylbenzisoselenazol-3(2*H*)-ones inactivated completely PL^pro^Cov2 in concentrations equal to 20 μM (Fig. 5c,d). In the case of PL^pro^SARS the range of inhibition was from 50 to 100% (see Fig. 5c,d).

The experiment with 2 μM of the inhibitor led us to identify highly active ligands (see Fig. 5e,f). Two derivatives of ebselen with substitution by hydroxy (**1d**) or methoxy group (**1e**) inhibit CoV2 fully. The affinities of diselenide orthologs (**2d** and **2e**) were overall in the same range. In contrast, replacing phenyl in position 2 with less hydrophobic substituents, hydrogen (**1a**) or methyl (**1b**), was not beneficial, similar to substitution by methyl in the *para* position of the phenyl ring (**1c**). We observed a similar relationship for diselenides (Fig. 5c,d). All compounds were less active toward PL^pro^SARS. Only compounds that are substituted derivatives of ebselen and their diselenide orthologs showed significant inhibition in the concentration range of 20 μM.

The inhibitory potency was further investigated for the most significant ligands with PL^pro^CoV2 and we found *IC*_50_ values in the nanomolar range for **1d**, **1e**, **2d** and **2e** (Fig. 5g). All four compounds appeared to be very effective inhibitors of PL^pro^ from CoV2, with the *IC*_50_ constants in the nanomolar range, e.g., 236 nM for compound **1d** with a hydroxyl substitution in the *ortho* position of the phenyl ring.

**Figure 6:**
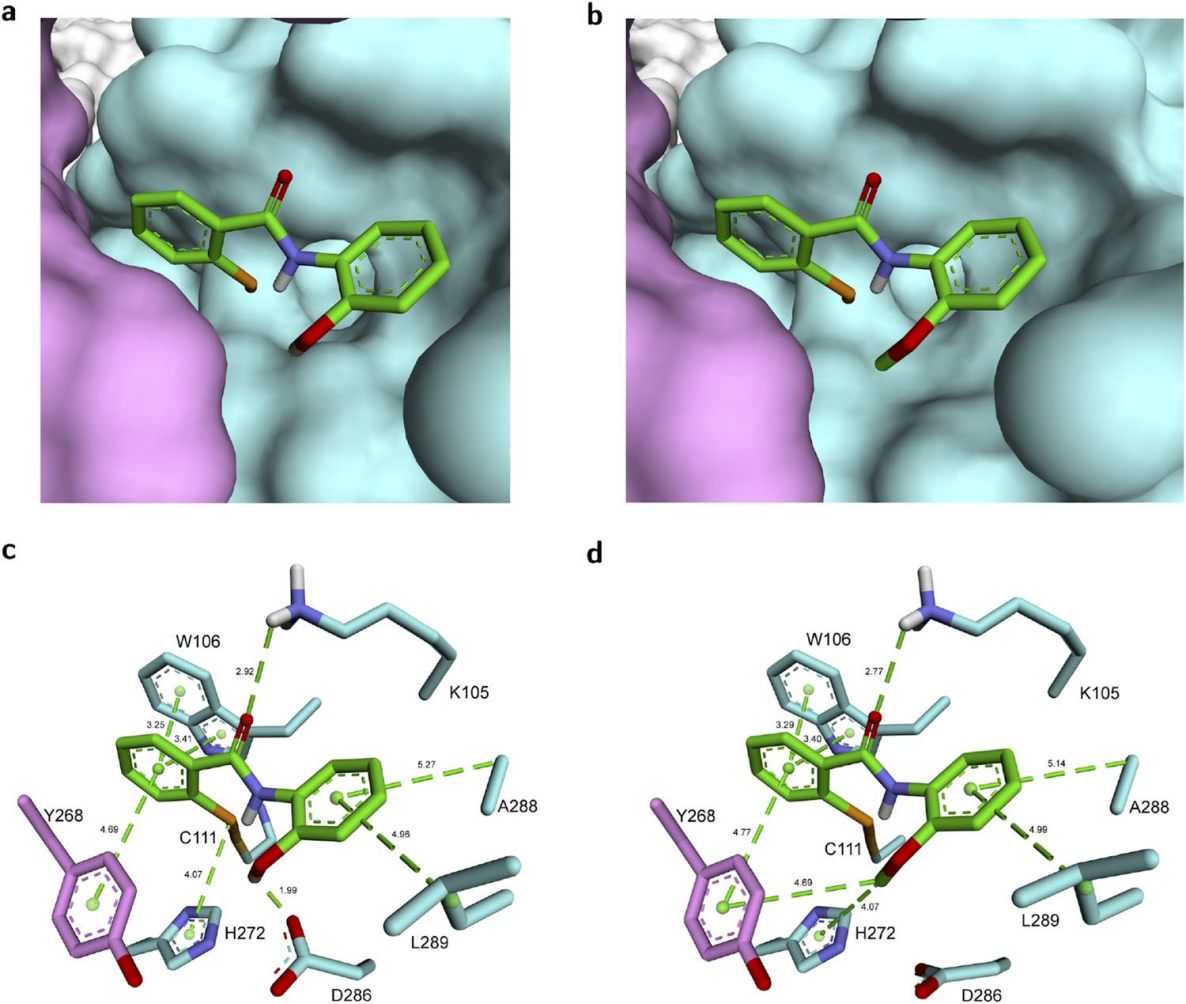
A model of the complex of hydroxyl and metoxyl derivatives with PL^pro^CoV2 shows additional interactions. (**a**,**b**) Molecular surface representation of the complex of **1d** (**a**) and **1e** (**b**) with PL^pro^CoV2 (PDB: 6W9C^37^). (**c**,**d**) Stick representation of the interactions between **1d** (**c**) and **1e** (**d**) and residues in the active site of PL^pro^CoV2 (PDB: 6W9C^37^).

The most significant results obtained in this study with the ebselen derivatives were further analyzed by molecular modeling (Fig. 6). The modeled interactions show similarities in the overall binding mode architecture compared with ebselen-PL^pro^CoV2 (Fig. 2c,d). However, introduction of hydroxyl (**1d**) or methoxy groups (**1e**) facilitate additional interactions with the active site (Fig. 6c,d). Similarly to ebselen complexes with enzyme, these selected derivatives occupy the same intersection between catalytic Cys111–His272–Asp286 triad and Trp106 and are wrapped by other Tyr268, Ala288 and Leu298 forming with them face-to-edge stacking and π-alkyl interactions, respectively (Fig. 6). Additionally, *Se*-phenyl and an indole from His272 forms π–π stacking interactions. In the case of the compound **1d** possessing a hydroxyl group, negatively charged oxygen atoms coordinate the carboxyl group from Asp286 (Fig. 6c). Whereas the methoxy group of the compound **1e** forms π-alkyl interactions with aromatic rings from His272 and Tyr268 (Fig. 6d).

## Discussion

The extensive research on developing new antiviral drugs for COVID-19 led to the identification of two potential target cysteine proteases that play a vital role in viral replication: M^pro^^35^ and PL^pro^^19^. PL^pro^, additionally to its essential role in virus replication, promotes the assembly of new viral particles within human cells^10,19^.

The identification of SARS-CoV-2 PL^pro^ as an essential viral enzyme^19^ offers a unique possibility for drug discovery. PL^pro^ is a protease that involves a strong nucleophilic cysteine thiol in a catalytic triad. The analysis and molecular modeling of the active site of PL^pro^ from coronaviruses SARS and CoV2 led us to conclude that small compounds with planar aromatic moieties, which are able to modify the cysteine residue of the active site, are promising candidate inhibitors. Selenoorganic compounds such as benzisoselenazol-3(2*H*)-ones **1** and bis(2-carbamoyl)phenyl diselenides **2** are perfect candidates due to the possibility of establishing the seleno-sulphur bond with a thiol of aminoacid cysteine residue.

In our study, we showed that ebselen, a selenoorganic drug with well-known anti-inflammatory, anti-atherosclerotic, and cytoprotective properties, which possesses a clean safety profile in human clinical trials, suppresses the enzyme PL^pro^CoV2 with *IC*_50_ approximately equal 2 μM. Moreover, we indicated that ebselen appeared to be an irreversible inhibitor of both PL^pro^CoV2 and PL^pro^SARS. However, it is four-fold weaker in the case of PL^pro^SARS. Additionally, we studied the binding mode of ebselen with PL^pro^SARS and PL^pro^CoV2 with the use of molecular modeling.

This observation reinforced our view regarding the mechanism of PL^pro^CoV2 enzyme inhibition by ebselen and aided in finding variants of the compound with further improved efficacy. Following the potential of ebselen as a ligand of PL^pro^, we tested eleven ebselen derivatives obtained by substitution/functionalization of the *N*-phenyl ring. The additional methoxy as well as hydroxy group in position *ortho* of the phenyl ring increased the inhibitory potency of ebselen by one order of magnitude and led us to identify most active inhibitors of PL^pro^CoV2 reported so far.

Moreover, we estimated parameters of kinetic constants and the catalytic efficiency of the processing of Ub-AMC by PL^pro^SARS and PL^pro^CoV2. Our results suggest that the capability to hydrolyze Ub-AMC is three times higher for PL^pro^SARS compared to PL^pro^CoV2. This observation strikingly aligns well with the fact that SARS is more aggressive than SARS-CoV-2 and leads to a faster development of disease.

In conclusion, we provided a further understanding of differences between SARS-CoV-1 and SARS-CoV-2 by analyzing PL^pro^ and identified very effective inhibitors of PL^pro^CoV2, with the *IC*_50_ constants in the nanomolar range. Our findings provide evidence that ebselen derivatives with an additional hydroxy or methoxy group are highly active inhibitors of the viral papain-like cysteine proteases (PL^pro^) that are essential for SARS-CoV-1 and SARS-CoV-2 replication. We believe the discovery reported here may contribute to the development of anti-COVID-19 therapy.

## Material and methods

### General

Recombinant SARS-CoV-1 PL^pro^, SARS-CoV-2 PL^pro^ and Ubiquitin-AMC were purchased as 32, 11 and 250 μM solutions, respectively, from R&D Systems.

Ebselen (2-Phenyl-1,2-benzisoselenazol-3(2H)-one) is commercially available from Sigma Aldrich.

All compounds were obtained and fully characterized in previous studies^39^. Their purity and homogeneity were confirmed by HRMS and ^77^Se NMR (see Supplementary data).

Enzyme and inhibition assays were designed based on the procedures described in^14,16,39,40^.

### Enzyme assays

The enzymes were dissolved in a 50 mM Tris–HCl buffer containing DTT (2 mM), NaCl (100 mM) and 0.1 mg/mL albumin, at pH 7.5, and preincubated 30 min. Spectrofluorimetric measurements were performed in a 96-well plate format working at two wavelengths: excitation at 355 nm and emission at 460 nm. The release of the fluorophore was monitored continuously at the enzyme concentration of 10 nM. The linear portion of the progress curve was used to calculate velocity of hydrolysis^14,16,39,40^.

### Enzyme reactivation

Covalent modification of enzymes was confirmed by regaining of the enzymatic activity after dialysis. The enzymes were incubated with inhibitors for 60 min in the assay buffer. Then, the reaction mixture was placed in a dialysis membrane and immersed in 50 mM Tris–HCl buffer, at pH 7.5 containing NaCl (100 mM). Dialysis was carried out on for 24 h at 4 °C. The activity was checked after 1, 3, 6 and 24 h as described above. The untreated enzyme was used as a standard.

### Inhibition assay

The inhibitor was screened against recombinant PL^pro^SARS and PL^pro^CoV2 at 37 °C in the assay buffer as described above. The release of the fluorophore was monitored continuously. The linear portion of the progress curve was used to calculate the velocity. Each experiment was repeated at least three times and the results are presented as the average with standard deviation. For more details, please see the materials and methods section. For steady state measurement the enzymes were incubated for 60 min at 37 °C with an inhibitor before adding the substrate to the wells. Eight different inhibitor concentrations were used. Value of the concentration of the inhibitor that achieved 50% inhibition (*IC*_50_) was taken from the dependence of the hydrolysis velocity on the logarithm of the inhibitor concentration [I]^14,16,39,40^.

### Molecular modeling

Molecular modeling studies were performed using the Discovery Studio 2020 (Dassault Systemes BIOVIA Corp). The crystal structures of the SARS-CoV-1 and SARS-CoV-2 (PDB ID 2FE8^10^ and 6W9C^37^, respectively) with protons added (assuming the protonation state of pH 7.5) were used as the starting point for calculations of the enzyme complexed with ebselen. In case of models representing the binding mode after the covalent modification, the Cys112 residue in the active site of SARS-CoV-1 and the Cys111 residue in the active site of SARS-CoV-2 were modified through the manual attachment of an appropriate selenoorganic inhibitor molecule. The partial charges of all atoms were computed using the Momany-Rone algorithm. Minimization was performed using the Smart Minimizer algorithm and the CHARMm force field up to an energy change of 0.0 or RMS gradient of 0.01. Generalized Born model was applied. The nonbond radius was set to 14 Å.

## Supplementary Information

Supplementary Information

## Data Availability

The data generated during and/or analyzed during the current study are available from the corresponding author upon reasonable request.

## References

[1] Summary Table of SARS Cases by Country, 1 November 2002–7 August 2003. WHO: Geneva (August 15, 2003). http://www.who.int/csr/sars/country/2003_08_15/en/.

[2] de Groot RJ (2013). Middle east respiratory syndrome coronavirus (MERS-CoV): Announcement of the coronavirus study group. J. Virol..

[3] Zhou P (2020). A pneumonia outbreak associated with a new coronavirus of probable bat origin. Nature.

[4] Wu F (2020). A new coronavirus associated with human respiratory disease in China. Nature.

[5] WHO Novel Coronavirus (COVID-19) pandemic. https://www.who.int/emergencies/diseases/novel-coronavirus-2019. (Data obtained on October 21, 2020).

[6] Tong TR (2009). Drug targets in severe acute respiratory syndrome (SARS) virus and other coronavirus infections. Infect. Disord. Drug Targets.

[7] Prajapat M (2020). Drug targets for corona virus: A systematic review. Indian J. Pharmacol..

[8] Rismanbaf A (2020). Potential treatments for COVID-19; a narrative literature review. Arch. Acad. Emerg. Med..

[9] Wu C (2020). Analysis of therapeutic targets for SARS-CoV-2 and discovery of potential drugs by computational methods. Acta Pharm. Sin. B..

[10] Báez-Santos M, St. John SE, Mesecar AD (2015). The SARS-coronavirus papain-like protease: Structure, function and inhibition by designed antiviral compounds. Antiviral Res..

[11] Harcourt BH (2004). Identification of severe acute respiratory syndrome coronavirus replicase products and characterization of papain-like protease activity. J. Virol..

[12] Ullrich S, Nitsche C (2020). The SARS-CoV-2 main protease as drug target. Bioorg. Med. Chem. Lett..

[13] Han YS (2005). Papain-like protease 2 (PLP2) from severe acute respiratory syndrome coronavirus (SARS-CoV): Expression, purification, characterization, and inhibition. Biochemistry.

[14] Lindner HA (2005). The papain-like protease from the severe acute respiratory syndrome coronavirus is a deubiquitinating enzyme. J. Virol..

[15] Devaraj SG (2007). Regulation of IRF-3-dependent innate immunity by the papain-like protease domain of the severe acute respiratory syndrome coronavirus. J. Biol. Chem..

[16] Barretto N (2005). The papain-like protease of severe acute respiratory syndrome coronavirus has deubiquitinating activity. J. Virol..

[17] Ratia K, Kilianski A, Baez-Santos YM, Baker SC, Mesecar AD (2014). Structural basis for the ubiquitin-linkage specificity and deISGylating activity of SARS-CoV papain-like protease. PLoS Pathog..

[18] Lindner HA (2007). Selectivity in ISG15 and ubiquitin recognition by the SARS coronavirus papain-like protease. Arch. Biochem. Biophys..

[19] Shin D (2020). Inhibition of papain-like protease PLpro blocks SARS-CoV-2 spread and promotes anti-viral immunity. Nature.

[20] Rut W (2020). Activity profiling and structures of inhibitor-bound SARS-CoV-2-PLpro protease provides a framework for anti-COVID-19 drug design. bioRxiv.

[21] Azad GK, Tomar RS (2014). Ebselen, a promising antioxidant drug: Mechanisms of action and targets of biological pathways. Mol. Biol. Rep..

[22] Chantadul V (2020). Ebselen as template for stabilization of A4V mutant dimer for motor neuron disease therapy. Commun. Biol..

[23] Hanavan PD (2015). Ebselen inhibits QSOX1 enzymatic activity and suppresses invasion of pancreatic and renal cancer cell lines. Oncotarget.

[24] Pietka-Ottlik M (2017). Synthesis of new alkylated and methoxylated analogues of ebselen with antiviral and antimicrobial properties. Arkivoc.

[25] Mukherjee S (2014). Ebselen inhibits hepatitis C virus NS3 helicase binding to nucleic acid and prevents viral replication. ACS Chem. Biol..

[26] Moriet K, al.  (1989). Phosphoroselenoate oligodeoxynucleotides: Synthesis, physico-chemical characterization, anti-sense inhibitory properties and anti-HIV activity. Nucleic Acids Res..

[27] Alexander V (2010). A new DNA building block, 4′-selenothymidine: Synthesis and modification to 4′-seleno-AZT as a potential anti-HIV agent. Org. Lett..

[28] Sancineto L (2015). Design and synthesis of diselenobisbenzamides (DISeBAs) as nucleocapsid protein 7 (NCp7) inhibitors with anti-HIV activity. J. Med. Chem..

[29] Pietka-Ottlik M, Potaczek P, Piasecki E, Mlochowski J (2010). Crucial role of selenium in the virucidal activity of benzisoselenazol-3 (2H)-ones and related diselenides. Molecules.

[30] Al-Smadi M, Al-Momani F (2008). Synthesis, characterization and antimicrobial activity of new 1, 2, 3-selenadiazoles. Molecules.

[31] Garland M (2020). The clinical drug ebselen attenuates inflammation and promotes microbiome recovery in mice after antibiotic treatment for CDI. Cell Rep. Med..

[32] Martini F (2019). A multifunctional compound ebselen reverses memory impairment, apoptosis and oxidative stress in a mouse model of sporadic Alzheimer’s disease. J. Psychiatr. Res..

[33] Gopalakrishna R, Gundimeda U, Chen ZH (1997). Cancer-preventive selenocompounds induce a specific redox modification of cysteine-rich regions in Ca(2+)-dependent isoenzymes of protein kinase. Arch. Biochem. Biophys..

[34] Azad GK (2014). Ebselen induces reactive oxygen species (ROS)-mediated cytotoxicity in Saccharomyces cerevisiae with inhibition of glutamate dehydrogenase being a target. FEBS Open Bio..

[35] Jin Z (2020). Structure of Mpro from COVID-19 virus and discovery of its inhibitors. Nature.

[36] Báez-Santos YM (2014). X-ray structural and biological evaluation of a series of potent and highly selective inhibitors of human coronavirus papain-like proteases. Med. Chem..

[37] Osipiuk, J. et al. The crystal structure of papain-like protease of SARS CoV-2. 10.2210/pdb6W9C/pdb.

[38] Tomczak JM, Węglarz-Tomczak E (2019). Estimating kinetic constants in the Michaelis–Menten model from one enzymatic assay using Approximate Bayesian Computation. FEBS Lett..

[39] Weglarz-Tomczak E (2016). Identification of methionine aminopeptidase 2 as a molecular target of the organoselenium drug ebselen and its derivatives/analoques: Synthesis, inhibitory activity and molecular modeling study. Bioorg. Med. Chem. Lett..

[40] Weglarz-Tomczak E (2013). An integrated approach to the ligand binding specificity of Neisseria meningitidis M1 alanine aminopeptidase by fluorogenic substrate profiling, inhibitory studies and molecular modelling. Biochimie.

