# Supplementary material for: Identification of ebselen and its analogues as potent covalent inhibitors of papain-like protease from SARS-CoV-2
Source: Sci Rep. 2021 Feb 11;11:3640. doi: 10.1038/s41598-021-83229-6 (PMC7878891; doi:10.1038/s41598-021-83229-6)
Supplement: Supplementary file 1 — Supplementary Information [file 41598_2021_83229_MOESM1_ESM.docx]

Supplementary Data
for

Identification of ebselen and its analogues as potent covalent inhibitors of papain-like protease from SARS-CoV-2

Ewelina Węglarz-Tomczak^1^, Jakub M. Tomczak^2^, Michał Talma^3^, Małgorzata Burda-Grabowska^3,4^, Mirosław Giurg^4^, Stanley Brul^1^

^1^Swammerdam Institute for Life Sciences, Faculty of Science, University of Amsterdam, the Netherlands

^2^Department of Computer Science, Vrije Universiteit Amsterdam, the Netherlands

^3^Department of Bioorganic Chemistry, Faculty of Chemistry, Wroclaw University of Science and Technology, Poland

^4^Department of Organic and Medicinal Chemistry, Faculty of Chemistry, Wroclaw University of Science and Technology, Wrocław, Poland

Corresponding authors:

[ewelina.weglarz.tomczak@gmail.com](mailto:ewelina.weglarz.tomczak@gmail.com) and [s.brul@uva.nl](mailto:s.brul@uva.nl)


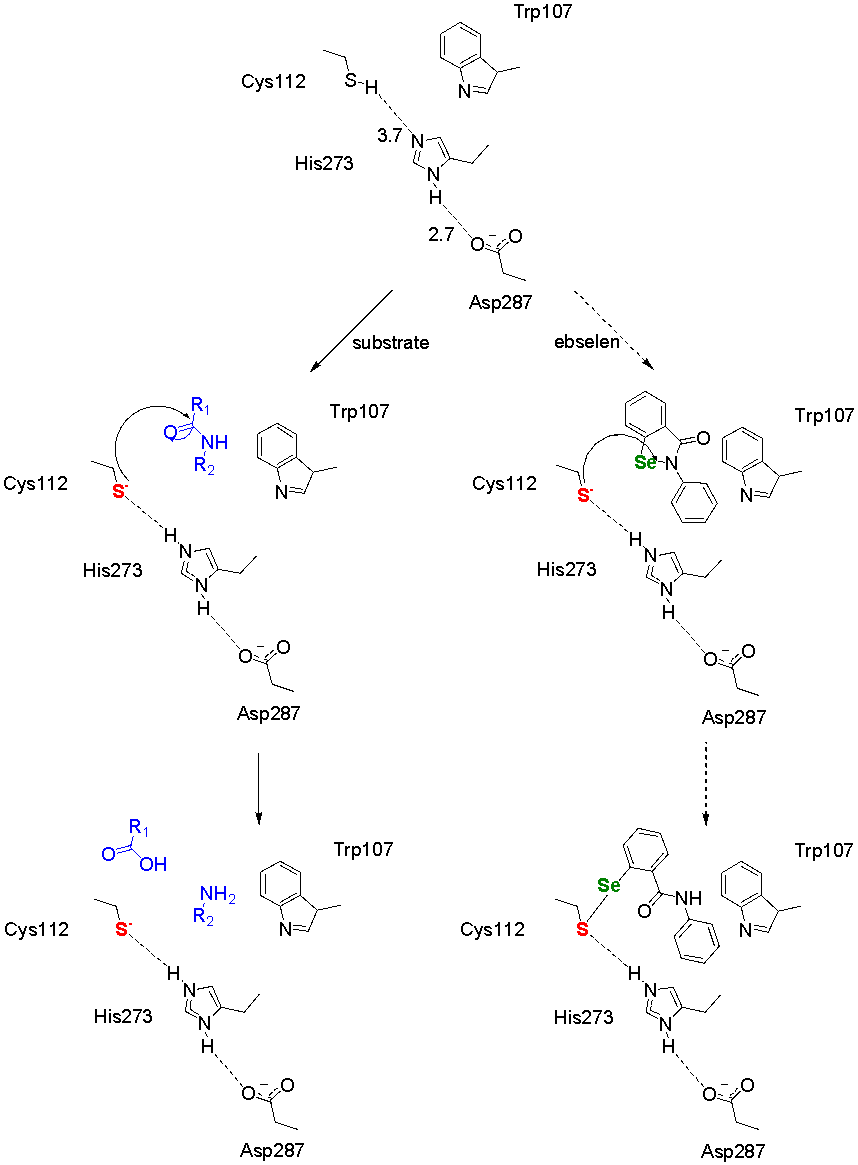


**Fig. 1.** Analysis of the active site, mechanism of the catalysis (left) and possible inhibition by ebselen (right) of coronaviruses PL^pro^ based on the active site of PL^pro^SARS [1].


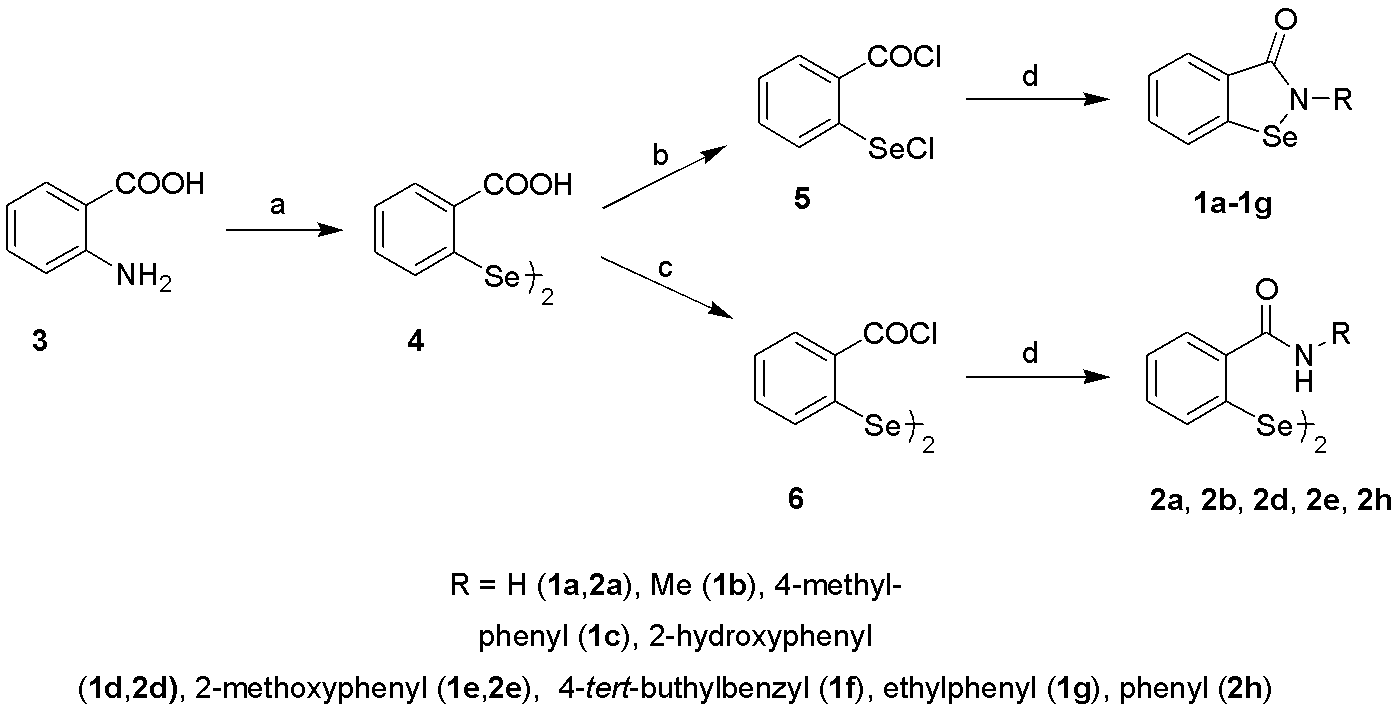


**Fig. 2.** Synthesis of the benzisoselenazol-3(2*H*)-ones **1a**–**1g** and bis(2-carbamoyl)phenyl diselenides **2a**, **2b**, **2d**, **2e**, **2h**. Reagents and conditions: a) i) HCl, H_2_O ii) NaNO_2_ -7–7°C, iii) Na_2_Se_2_,-5–+5°C, next rt 20h, iv) HCl; gentle reflux, and rt for 20h; b) 7 eqv. SOCl_2_, DMF, benzene, reflux, 3.5h; c) 3.5eqv SOCl_2_, DMF, benzene, reflux; d) RNH_2_, Et_3_N, MeCN, or RNH_2_, MeCN [2].

**Synthesis**

The compounds considered in this paper were fully characterized in the previous studies [2-8]. Their purity and homogeneity were confirmed by HRMS and ^77^Se NMR.

**1,2-Benzisoselenazol-3(2H)-one (1a)**

^77^Se NMR (115 MHz, DMSO-d_6_): δ 808.35 ppm. HRMS (TOF MS ESI): m/z for C_7_H_5_NOSe+H^+^ calculated: 199.9615; found: 199.9606.

**2-Methyl-1,2-benzisoselenazol-3(2H)-one (1b)**

^77^Se NMR (115 MHz, DMSO-d_6_): δ 877.08 ppm. HRMS (TOF MS ESI): m/z for C_8_H_7_N_2_OSe+H^+^ calculated: 213.9771; found: 213.9762.

**2-(4-Methylphenyl)-1,2-benzisoselenazol-3(2H)-one (1c)**

^77^Se NMR (115 MHz, DMSO-d_6_): δ 925.64 ppm. HRMS (TOF MS ESI): m/z for C14H11NOSe+Na+ calculated: 311.9904; found: 311.9906.

**2-(2-Hydroxyphenyl)-1,2-benzisoselenazol-3(2H)-one (1d)**

^77^Se NMR (115 MHz, DMSO-d_6_): δ 930.02 ppm. HRMS (TOF MS ESI): m/z for C_13_H_9_NO_2_Se+H^+^ calculated: 291.9877; found: 291.9984.

**2-(2-Methoxyphenyl)-1,2-benzisoselenazol-3(2H)-one (1e)**

^77^Se NMR (115 MHz, DMSO-d_6_): δ 946.31 ppm. HRMS (TOF MS ESI): m/z for C_14_H_11_NO_2_Se+H^+^ calculated: 306.0034; found: 306.0041.

**2-(4-t-Butylbenzyl)-1,2-benzisoselenazol-3(2H)-one (1f)**

^77^Se NMR (115 MHz, DMSO-d_6_): δ 867.86 ppm. HRMS (TOF MS ESI): m/z for C_18_H_19_NOSe+H^+^ calculated: 346.0711; found: 346.0701.

**2-Phenylethyl-1,2-benzisoselenazol-3(2H)-one (1g)**

^77^Se NMR (115 MHz, DMSO-d_6_): δ 875.57 ppm. HRMS (TOF MS ESI): m/z for C_15_H_13_NOSe+H^+^ calculated: 304.0241; found: 304.0337.

**Bis[2-(2-hydroxyphenylcarbamoyl)phenyl] diselenide (2d)**

^77^Se NMR (115 MHz, DMSO-d_6_): δ 458.63 ppm. HRMS (TOF MS ESI): m/z for C_26_H_20_N_2_O_4_Se_2_+Na. calculated: 606.9657; found: 606.9700.

**Bis[2-(2-methoxyphenylcarbamoyl)phenyl] diselenide (2e)**

^77^Se NMR (115 MHz, DMSO-d_6_): δ 455.29 ppm. HRMS (TOF MS ESI): m/z for C_28_H_24_N_2_O_4_Se_2_+Na^+^ calculated: 634.9970; found: 634.9996.

**Bis(2-phenylcarbamoyl)phenyl diselenide (2h)**

^77^Se NMR (115MHz, DMSO-d6): δ 439.78ppm. HRMS (TOF MS ESI): m/z for C26H20N2O2Se2+Na+calculated: 574.9752; found: 574.9752.

**Approximate Bayesian Computation for estimating kinetic constants**

The full description of the Approximate Bayesian Computation (ABC) for estimating kinetic constants in the Michaelis-Menten model is available in the literature [9].

The goal of the method is to find values of kinetic constants *k*_cat_ and *K*_M_ that minimize the Euclidean distance between real measurements and signals generated by the Michaelis-Menten. The method consists of the following steps:

1. Sample new values of parameters *k*_cat_ and *K*_M_.
2. Simulate data using the Michaelis-Menten model. This step requires applying a numerical method for solving ordinary differential equations (e.g., the Runge-Kutta methods).
3. Calculating the distance between the real measurements and the generated data. If the distance is smaller than some assumed threshold, the values of parameters are accepted. Otherwise, the values of parameters are rejected.
4. If the stopping criterion is not met (e.g., the number of repetitions), go to step 1. Otherwise stop the procedure and calculate mean and standard deviation of all accepted values of *k*_cat_ and *K*_M_.

**References**

1. Báez-Santos M., St. John S. E. & Mesecar A. D. The SARS-coronavirus papain-like protease: Structure, function and inhibition by designed antiviral compounds. *Antiviral Res.* **115**, 21–38 (2015).
2. Weglarz-Tomczak, E. et al. Identification of methionine aminopeptidase 2 as a molecular target of the organoselenium drug ebselen and its derivatives/analogues: Synthesis, inhibitory activity and molecular modeling study. *Bioorg. Med. Chem. Lett.* **26**, 5254–5259 (2016).
3. Młochowski, J., Kloc, K., Syper, L., Inglot, A. D. & Piasecki, E. Aromatic and azaaromatic diselenides, benzisoselenazolones and related compounds as immunomodulators active in humans: synthesis and properties, *Liebigs Ann. Chem*. 12, 1239-1244 (1993).
4. Młochowski, J. et al. Synthesis and properties of 2‐carboxyalkyl‐1, 2‐benzisoselenazol‐3 (2H)‐ones and related organoselenium compounds as nitric oxide synthase inhibitors and cytokine inducers, *Liebiegs Ann. Chem*., 1751-1755, **11** (1996).
5. Chang, T.-C., Huang, M.-L., Hsu, W.-L., Hwang, J.-M. & Hsu, L.-Y. Synthesis and biological evaluation of ebselen and its acyclic derivatives, *Chem. Pharm. Bull.*, **51**, 1413 (2003).
6. Welter, A., Fischer, H., Christiaens, L.; Wendel, A.; Etschenberg, E. DE 3513070 (1986).
7. Welter, A., Leyck, S. & Etschenberg, E. DE 3407511 (1985).
8. Balkrishna, S. J., Bhakuni, B. S., Chopra, D. & Kumar, S. Cu-catalyzed efficient synthetic methodology for ebselen and related Se− N heterocycles. *Org. Lett.*, **12**, 5394-5397 (2010).
9. Tomczak, J.M. & Węglarz-Tomczak, E. Estimating kinetic constants in the Michaelis-Menten model from one enzymatic assay using Approximate Bayesian Computation. *FEBS Lett.* **593**, 2742-2750 (2019).
